# Use of artificial intelligence in the analysis of digital videos of invasive surgical procedures: scoping review

**DOI:** 10.1093/bjsopen/zraf073

**Published:** 2025-07-17

**Authors:** Anni King, George E Fowler, Rhiannon C Macefield, Hamish Walker, Charlie Thomas, Sheraz Markar, Ethan Higgins, Jane M Blazeby, Natalie S Blencowe

**Affiliations:** National Institute for Health Research Bristol Biomedical Research Centre (Surgical and Orthopeadic Innovation Theme), University of Bristol & Centre for Surgical Research, Bristol Medical School: Population Health Sciences, University of Bristol, Bristol, UK; National Institute for Health Research Bristol Biomedical Research Centre (Surgical and Orthopeadic Innovation Theme), University of Bristol & Centre for Surgical Research, Bristol Medical School: Population Health Sciences, University of Bristol, Bristol, UK; National Institute for Health Research Bristol Biomedical Research Centre (Surgical and Orthopeadic Innovation Theme), University of Bristol & Centre for Surgical Research, Bristol Medical School: Population Health Sciences, University of Bristol, Bristol, UK; National Institute for Health Research Bristol Biomedical Research Centre (Surgical and Orthopeadic Innovation Theme), University of Bristol & Centre for Surgical Research, Bristol Medical School: Population Health Sciences, University of Bristol, Bristol, UK; National Institute for Health Research Bristol Biomedical Research Centre (Surgical and Orthopeadic Innovation Theme), University of Bristol & Centre for Surgical Research, Bristol Medical School: Population Health Sciences, University of Bristol, Bristol, UK; Nuffield Department of Surgical Sciences, Oxford University Hospitals, Oxford, UK; School of Medicine, University of Sunderland, Sunderland, UK; National Institute for Health Research Bristol Biomedical Research Centre (Surgical and Orthopeadic Innovation Theme), University of Bristol & Centre for Surgical Research, Bristol Medical School: Population Health Sciences, University of Bristol, Bristol, UK; National Institute for Health Research Bristol Biomedical Research Centre (Surgical and Orthopeadic Innovation Theme), University of Bristol & Centre for Surgical Research, Bristol Medical School: Population Health Sciences, University of Bristol, Bristol, UK

**Keywords:** surgery, operative, procedures, video, artificial intelligence, review

## Abstract

**Introduction:**

Surgical videos are a valuable data source, offering detailed insights into surgical practice. However, video analysis requires specialist clinical knowledge and takes considerable time. Artificial intelligence (AI) has the potential to improve and streamline the interpretation of intraoperative video data. This systematic scoping review aimed to summarize the use of AI in the analysis of videos of surgical procedures and identify evidence gaps.

**Methods:**

Systematic searches of Ovid MEDLINE and Embase were performed using search terms ‘artificial intelligence’, ‘video’, and ‘surgery’. Data extraction included reporting of general study characteristics; the overall objective of AI; descriptions of data sets, AI models, and training; methods of data annotation; and measures of accuracy. Data were summarized descriptively.

**Results:**

In all, 122 studies were included. More than half focused on gastrointestinal procedures (75 studies, 61.5%), predominantly cholecystectomy (47, 38.5%). The most common objectives were surgical phase recognition (40 studies, 32.8%), surgical instrument recognition (28, 23.0%), and enhanced intraoperative visualization (23, 18.9%). Of the studies, 79.5% (97) used a single data set and most (92, 75.4%) used supervised machine learning techniques. There was considerable variation across the studies in terms of the number of videos, centres, and contributing surgeons. Forty-seven studies (38.5%) did not report the number of annotators, and details about their experience were frequently omitted (102, 83.6%). Most studies used multiple outcome measures (67, 54.9%), most commonly overall or best accuracy of the AI model (67, 54.9%).

**Conclusion:**

This review found that many studies omitted essential methodological details of AI training, testing, data annotation, and validation processes, creating difficulties when interpreting and replicating these studies. Another key finding was the lack of large data sets from multiple centres and surgeons. Future research should focus on curating large, varied, open-access data sets from multiple centres, patients, and surgeons to facilitate accurate evaluation using real-world data.

## Teaser

There is a growing body of research investigating the ability of artificial intelligence to improve the quality of surgical healthcare, specifically via the interpretation of operative videos. This review summarizes the use of artificial intelligence to facilitate surgical video analysis.

## Introduction

Advances in camera technologies and minimally invasive surgery have eased the burden of intraoperative video recording and data transfer^1^. Despite this, there is considerable variation in how videos are captured and analysis undertaken, and only a minority of UK surgeons routinely record procedures^2^. Surgical videos are a valuable data source offering a comprehensive, unbiased insight into surgical practice. They provide an objective account of events in comparison to operative notes, which rely on surgeons’ recall and documentation^3,4^. However, reviewing and analysing surgical videos is resource intensive, subject to bias, and requires expert clinical knowledge. In recent years, artificial intelligence (AI) has been viewed as a catalyst for transforming healthcare practice. The term AI refers to technology that enables machines and software to undertake activities traditionally requiring human intelligence^5^.

There is a growing body of research investigating the ability of AI to improve the quality of surgical healthcare, specifically via the interpretation of diagnostic imaging such as radiography^6^, computed tomography scans^7^, magnetic resonance imaging^8^, and conventional photography^9^. More recently, the potential of AI for analysing surgical videos has been recognized, resulting in several recommendations regarding video annotation and data acquisition, transfer, storage, use, and governance^10,11^. AI has the potential to assist real-time operator guidance, facilitate error detection, optimize surgical workflow, and improve the efficiency of research and training^12,13^. Understanding methods to improve and streamline video analysis to ensure this valuable data is appropriately used is now paramount. The aim of this scoping review was to summarize the methods used to apply AI in the analysis of videos of surgical procedures and identify evidence gaps for future research.

## Methods

A predefined protocol was prepared^14^ in accordance with PRISMA extension for scoping reviews^15^. A scoping review was considered appropriate for this study given the need to determine the scope and volume of this emerging research area, as well as to provide an overview of the research. An invasive procedure was defined as one where deliberate access to the body is gained via an incision or percutaneous puncture^16^. Endoscopic procedures were excluded due to numerous existing reviews in this area^17–19^ and to limit findings to a manageable amount of data.

### Search strategy

A comprehensive search syntax was developed using text words and medical subject headings (MeSH) relating to three domains: ‘artificial intelligence’, ‘video’, and ‘surgery’. Search terms were designed to allow for the wide and diverse terminology used when describing AI applications, methods, and types of imaging (*Appendix S1*). Searches were conducted in OVID MEDLINE and Embase for articles published between 1 January 2012 and 22 November 2023.

### Eligibility criteria and study selection

Primary research studies involving living human participants and written in English were considered for eligibility. Videos of open and minimally invasive surgery (that is, laparoscopic, thoracoscopic, robotic) were included. Studies incorporating stills/frames from videos were included if the initial data set consisted of videos. Secondary research studies (that is, systematic reviews), case reports, case series, editorials, and conference abstracts were excluded.

Abstracts were screened for eligibility by at least two independent reviewers (A.K., G.E.F., and N.S.B.). The full text of potentially relevant articles was subsequently reviewed by at least two independent reviewers (A.K., G.E.F., N.S.B., H.W., and C.T.). Reference lists of included articles were examined to identify any additional relevant articles that may not have been identified via the electronic searches. A study-specific database was designed in RedCap software^20,21^ and piloted by members of the study team (A.K., G.E.F., and N.S.B.).

Data were extracted in the following categories: study characteristics (journal, year of publication, country of origin, modality of surgery, and specialty of interest); data set characteristics (data source, number of videos, surgeons and centres); input data features (size and nature of test and training sets, number and experience of annotators and software used); the main objective(s) of the AI; details of any comparators and/or validation methods; and reporting of any measures of accuracy of the AI model and their limitations.

### Quality appraisal

Quality appraisal was not undertaken due to the lack of availability of suitable tools. Although some AI-specific quality appraisal assessments exist, they are specifically for research into the diagnostic capabilities of AI and assessment of prediction model studies^22,23^.

## Results

**Fig. 1:**
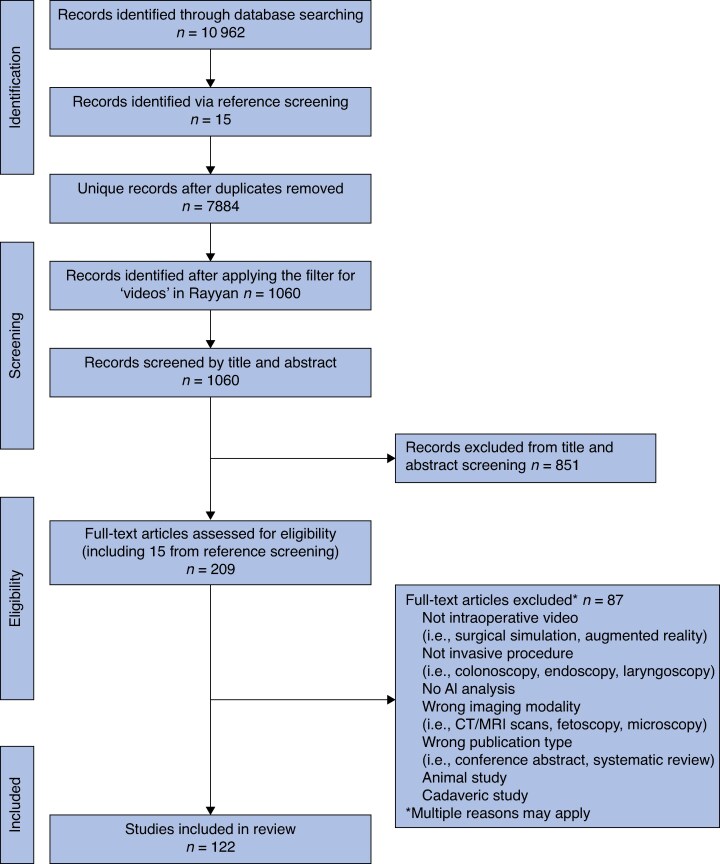
PRISMA flow diagram showing the screening process for article selection AI, artificial intelligence; CT, computed tomography; MRI, magnetic resonance imaging.

Searches identified 1060 papers for abstract screening. After the removal of duplicates and full text screening, 122^24–143^ full-text papers were included in the analysis (*Fig. 1*).

Most papers were published from 2020 onwards, with more than half focusing on gastrointestinal procedures, predominantly cholecystectomy. Only four studies incorporated the analysis of open surgical procedures (*Appendix S2*).

### Data set characteristics

*Table 1* details the key characteristics of the included data sets. Almost one-third of studies used open-source, publicly available data sets (full details on open-source data sets used in studies are provided in *Appendix S3*). Most studies used Cholec80, a data set of 80 laparoscopic cholecystectomy videos performed by 13 surgeons. Data sets comprised video clips, still images/frames, or a mix of both. Most studies used a single data set of ≤ 100 videos (median 70 videos (i.q.r. 26–122)). The largest data set (2906 videos) was from a study aiming to train AI models to identify and classify suturing gestures during robotic prostatectomy^24^. The number of surgeons and centres contributing to the data set was often not reported. One study attempting to develop AI models to identify safe and dangerous zones of dissection during laparoscopic cholecystectomy procured videos from 153 surgeons from 136 institutions across 37 countries^48^.

**Table 1:** Characteristics of the included data sets from 122 studies

Characteristic	No. of studies
**Source**	
Open source (publicly available)	39
Primary data (collected during study)	36
Pre-existing collection of videos	34
Multisource (combination of open/primary/pre-existing)	10
Not specified	3
**No. of data sets**	
1	97
2	17
3	7
Unclear	1
**No. of videos in data set**	
1–50	50
51–100	33
101–200	12
>201	19
Not reported	8
Type and amount of training data*	
Videos	
1–50	35
51–100	8
101–200	8
>200	8
Stills	
<500	6
501–2500	9
>2500	11
Unclear	1
Videos + stills	4
Not specified	19
No training data set	13
Type and amount of test data*	
Videos	
1–50	51
51–100	5
101–200	3
>200	4
Stills	
<500	13
501–2500	3
>2500	5
Unclear	1
Videos + stills	4
Not specified	20
No test data set	12
No. surgeons contributing to data set*	
<5	16
5–50	26
>50	3
Not reported	77
No. of centres contributing to data set*	
<5	62
5–50	4
>50	2
Not reported	54
**Patient consent for video recording**	
N/A (open source data set)	38
Yes	38
Not stated	37
No	9

^*^Data are reported on only the first data set (that is, if multiple data sets are used this not reported; 17 studies). N/A, not applicable.

### Input data features

Most studies used test data sets comprised of videos (often ≤ 50). Of those using stills/images, most used ≤ 500 stills/images. Almost three-quarters of studies used a training data set (59 videos; 27 stills/frames). Although video training data sets were often small, with many studies using ≤ 50 videos, there was considerable variation across studies (median 40 videos (i.q.r. 27–103)). The stills/frames training sets were similarly variable, with most using ≤ 2500 stills (median 1712 stills (i.q.r. 560–5000)). One study, which aimed to build a large annotated data set of laparoscopic colorectal procedures for automated recognition of surgical phase, action, and tool presence, used 66,001,529 annotated images (from 240 videos) for training purposes and 16,621,569 images for testing^49^.

Most studies did not report which software they used to annotate the videos or stills, or the number of annotators and their relevant experience (*Appendix S4*).

### AI objectives and models used

The most stated objectives included surgical phase recognition, instrument recognition and tracking, enhanced intraoperative visualization, assessment of surgical skill, and identification of anatomy (*Table 2*). Most studies used supervised learning, predominantly convolutional neural networks (CNN) and artificial neural networks (ANN).

**Table 2:** Description of AI models used and their objectives

AI model	Objective of AI application (no. of studies)									
	Keyframe extraction (*n* = 2 studies)	Skill assessment (*n* =13 studies)	Surgical phase/workflow recognition (*n* =40 studies)	Enhanced intraoperative visualization and safety (*n* =23 studies)	Identification of procedure type (*n* = 2 studies)	Identification of procedural duration (*n* = 2 studies)	Instrument recognition (including tracking; *n* = 28 studies)	Identification of anatomy (*n* = 13 studies)	Task/gesture identification (*n* = 4 studies)	Other objective* (*n* =5 studies)
ANN (*n* = 23)	0	3	7	2	0	0	4	6	1	0
SVM (*n* = 10)	0	2	2	1	1	0	1	2	1	0
CNN (*n* = 68)	1	7	29	5	1	2	19	7	4	2
Deep CNN (*n* = 11)	0	1	3	5	0	0	2	1	0	2
LRA (*n* = 1)	0	1	0	0	0	0	0	0	0	0
Decision tree (*n* = 1)	0	0	0	1	0	0	0	0	0	1
Bayesian networks (*n* = 3)	1	0	0	0	0	0	1	0	0	1
HMM (*n* = 6)	0	0	5	1	0	0	0	0	0	0
LSTM (*n* = 15)	0	1	9	0	1	1	3	0	0	0
Not specified (*n* = 5)	0	1	1	2	0	0	1	0	0	0
Recurrent CNN (*n* = 4)	0	1	1	1	0	0	1	0	0	0
GNN (*n* = 2)	0	0	1	0	0	0	0	1	0	0
Other model† (*n* = 4)	0	0	0	2	0	0	1	1	0	2

Total counts may exceed the total number of studies because some studies used multiple models (29 studies) and/or multiple objectives (11 studies). *Other objectives include smoke detection, identifying out-of-body views, prediction of postoperative recovery, education and teaching, detection of surgical gauze, and automated mining of web-based videos of surgery. †Other models include rigid part mixtures model, adaptive deformation solidification module, OoBNet, and invertible neural networks. AI, artificial intelligence; ANN, artificial neural networks; SVM, support vector machines; CNN, convolutional neural networks; LRA, logistic regression analysis; HMM, hidden Markov models; LSTM, long short-term memory; GNN, graph neural network.

Less than one-third of studies reported the use of a comparator during model evaluation, most of which were comparisons with other state-of-the-art AI models. Most studies did not report the use of processes to establish inter-rater reliability or cross-checking methods. Over three-quarters of studies undertook validation testing using an internal data set, and AI analysis and testing were rarely undertaken in real time (*Appendix S5*).

### Outcome measures and limitations

Most studies reported multiple outcome measures, most commonly accuracy (overall and/or best); precision, recall and F1 score, sensitivity; and predictive values (*Appendix S5*). Overall and/or best accuracy across the studies is summarized in *Appendix S6*, along with any reported model limitations. The most described limitations were small numbers of videos, stills, surgeons, or centres contributing to the data set, or that the data set was limited to a single surgical procedure.

## Discussion

This scoping review of the use of AI for the analysis of videos of invasive surgical procedures summarized information from 122 eligible studies published between 2012 and 2023. It found that most of the included studies were preclinical studies and focused on building annotated data sets for identifying surgical phase, enhancing visualization, and instrument recognition. Several reporting issues were identified, with many studies omitting essential methodological details. Key information about the content of data sets was also frequently omitted. This can affect the transparency and generalizability of study findings, and their reproducibility.

Several guidelines have been published relating to the use of AI in healthcare and clinical trials^144,145^. A recent systematic review identified 26 reporting guidelines and checklists^146^, of which 8 include recommendations for reporting on annotation methods^147–154^. For example, one suggested that the number of annotators, their qualifications, software used, whether annotations were done independently, and any inter-rater and intra-rater-reliability testing should all be reported^150^. This abundance of available guidance may be adding to the complexity around reporting; simple and unified guidance would therefore be beneficial.

The preponderance of data sets from a single centre creates a lack of diversity in the demography of surgeons and patients and potentially procedural complexity, which has implications for the capabilities and accuracy of AI models. A lack of diversity in demographic characteristics can lead to unknown biases reflected in data sets, which may unintentionally subsequently embed within AI models^155^. One way of improving this would be to facilitate the creation of more large, high-quality, annotated, open-source data sets. Even Cholec80 has potential limitations, despite its widespread use. The videos display mostly linear workflows and may therefore be devoid of potential real-world challenges, such as additional phases and intraoperative complications^156^. Such exemplar videos usually also exclude occlusions, insufficient lighting, and other technical hindrances found in everyday practice. It would be desirable to capture the complexity of real-world surgical scenarios and record data under less controlled conditions, such as the ENDO-Vis 2022 SAR-RARP50 challenge, whereby a specific subtask was to investigate model performance when applied to real-world data with challenging scenarios, including technical conditions^157^.

Some limitations are acknowledged. Despite searching two large databases, relevant studies may still have been missed due to indexing variations and by excluding studies not published in English. Specialist technical databases may also have been missed. Studies included in the review are also limited to video analysis of invasive surgical procedures, meaning that information from other procedures (for example, endoscopy or percutaneous procedures) was excluded. Finally, the search strategy ended in November 2023, meaning that more recent studies were missed.

Coupled with emerging state-of-the-art camera technologies, there is little doubt that AI has the potential to transform the surgical pathway. The adoption of AI into surgery has been slow compared with industry sectors, and empirical studies exploring AI to automate the analysis of surgical videos are still emerging. This review demonstrated variation in reporting and the omission of key methodological details, which only add to difficulties in interpretability, transparency, and replicability. The provision and uptake of reporting guidance would help interpret results, understand their reliability, guide further developments, and explain when things go wrong. Such guidance could also extend to best practice for data sets, potentially including the incorporation of open-source video data sets, to ensure model training is capable of evaluation on future real-world data. Work is now needed to facilitate video capture, transfer, and storage within UK National Health Service infrastructures and to encourage the uptake of surgical video recordings.

## Supplementary Material

zraf073_Supplementary_Data

## Data Availability

All relevant data are within the paper and its *Supplementary material*.
