# Supplementary material for: Use of artificial intelligence in the analysis of digital videos of invasive surgical procedures: scoping review
Source: BJS Open. 2025 Jul 17;9(4):zraf073. doi: 10.1093/bjsopen/zraf073 (PMC12268333; doi:10.1093/bjsopen/zraf073)
Supplement: zraf073_Supplementary_Data [file zraf073_supplementary_data.docx]

**Title**

**The use of Artificial Intelligence in the analysis of digital videos of invasive surgical procedures: scoping review**

Authors

Anni King^1^, [anni.king@bristol.ac.uk*](mailto:anni.king@bristol.ac.uk*)

George Fowler^1^, [George.Fowler@bristol.ac.uk*](mailto:George.Fowler@bristol.ac.uk*)

Rhiannon C Macefield^1^, [R.Macefield@bristol.ac.uk](mailto:R.Macefield@bristol.ac.uk)

Hamish Walker^1^, [hamish.walker2@nhs.net](mailto:hamish.walker2@nhs.net)

Charlie Thomas^1^, [sm18548@bristol.ac.uk](mailto:sm18548@bristol.ac.uk)

Ethan Higgins^3^ [bh95uj@student.sunderland.ac.uk](mailto:bh95uj@student.sunderland.ac.uk)

Sheraz Markar^2^, [sheraz.markar@nds.ox.ac.uk](mailto:sheraz.markar@nds.ox.ac.uk)

Jane M Blazeby^1^, [J.M.Blazeby@bristol.ac.uk](mailto:J.M.Blazeby@bristol.ac.uk)

Natalie S Blencowe^1^, Natalie.Blencowe@bristol.ac.uk**

*Joint first author

**Senior author

**AUTHOR AFFILIATIONS**

^1^ National Institute for Health Research Bristol Biomedical Research Centre (Surgical Innovation Theme), Centre for Surgical Research, Bristol Medical School: Population Health Sciences, University of Bristol, Bristol, UK. BS8 2PS.

^2^ Nuffield Department of Surgery, Oxford University Hospitals

^3^ School of Medicine, University of Sunderland

**Corresponding author.** Anni King, [anni.king@bristol.ac.uk](mailto:anni.king@bristol.ac.uk), https://**orcid**.org/0000-0002-4489-0864

**Supplementary Materials - Index**

| **Supplementary Appendixes** |  |
| --- | --- |
| **Appendix 1:** Search strategy for OVID Medline and Embase | *pag. 3* |
| ***Appendix 2:*** *Characteristics of included publications*  ***Appendix 3:*** *Open-source datasets*  **Appendix 4:** *Details of data annotation*  **Appendix 5:** *Characteristics of machine learning and outcome measures (i.e., use of* comparators, methods of internal/external model validation and if any assessments were undertaken in real-time).  **Appendix 6:** Accuracy of AI performance and reported limitations | *pag. 4-5*  *pag. 6*  *pag. 7*  *pag. 8*  *pag. 9-14* |
|  |  |
|  |  |
|  |  |
|  |  |

**Supplementary Methods**

**Appendix 1. Search strategy for OVID Medline and Embase**

1. ((("Machine Learning" [Mesh] OR "Artificial Intelligence"[Mesh] OR "Natural Language Processing"[Mesh] OR "Neural Networks (Computer)"[Mesh] OR "Support Vector Machine"[Mesh] OR Machine learning.tw OR Artificial Intelligence.tw OR Naive Bayes.tw OR bayesian learning.tw OR Neural network.tw OR Neural networks.tw OR Natural language processing.tw OR support vector*.tw OR random forest*.tw OR deep learning.tw OR deep-learning.tw OR machine intelligence.tw OR computational intelligence.tw OR computer reasoning.tw OR (Decision Tree Analysis) OR reinforcement learning OR reinforcement-learning OR Boltzmann machine* OR "long short-term memory" OR "gated recurrent unit" OR "rectified linear unit" OR autoencoder OR backpropagation OR "multilayer perceptron" OR convnet OR "convolutional learning")))

AND

1. Operating rooms/ OR surgical procedures, operative/ OR Surgery, computer-assisted/ or “Surg*”.tw OR “operating theatre”.tw OR “operating room”.tw or “intraoperativ*”.tw

AND

1. (Photography/ or Video Recording/ or Image Processing, Computer-Assisted/ or Imaging, Medical/ or Medical Imaging/) or ("visual" or "digital imag*" or "camera" or "phot*" or "video" or "imaging" or "scan").tw.

AND

1. Limit to date range 2012-current and publication language=English

NOT

1. Comment [Publication Type] OR editorial [Publication Type] OR letter [Publication Type]

**Supplementary Results**

**Appendix 2.** *Characteristics of included publications*

| **Characteristic** |  | **(n=122)** |
| --- | --- | --- |
| Year of Publication | 2020-present  2015-2019  2012-2014 | 91  30  1 |
| Country of Origin | Asia  Europe  United States of America  United Kingdom  Australasia  Canada | 49  48  18  5  1  1 |
| Surgical Specialty | Upper Gastrointestinal   - *Cholecystectomy* - *Gastrectomy (including sleeve)* - *Oesophagectomy* - *Gastric bypass* - *Hepatectomy*   Lower Gastrointestinal   - *Sigmoidectomy* - *Transanal total mesorectal excision (TaTME)* - *Rectopexy surgery* - *Radical colectomy* - *Colorectal resection*   General   - *Hernia Repair*   Urology   - *Radical prostectomy* - *Partial nephrectomy* - *Radical cystectomy*   Obstetrics & Gynaecology   - *Endometriosis treatment* - *Hysterectomy*   Cardio-thoracics  *- Pulmonary valve replacement*  Trauma and Orthopeadics  *- Arthroscopic rotator cuff surgery*  *- Biportal endoscopic spine surgery*  *- Total knee replacement*  SEndocrine  *- Thyroid surgery*  *- Pituitary surgery*  Unreported/undefined  Multiple | 47  8  4  2  2  6  2  1  1  2  6  4  2  1  1  1  1  1  1  1  2  1  1  7  17 |
| Modality of surgery | Laparoscopic  Robotic-assisted  Laparoscopic and robotic  Open  Laparoscopic, robotic and open | 90  17  10  4  1 |
| Ethics Approval | Yes  Not stated  No (not required) | 55  45  22 |
| Funding | Yes   - *Non-commercial (charity, government, university)* - *Commercial* - *Both*   Unclear  Not stated  No funding | 88  76  8  3  1  22  12 |

**Appendix 3. Open-source datasets**

| **Dataset** | **Description** | **No. papers** |
| --- | --- | --- |
| **Bypass40** | 40 videos of Laparoscopic Roux-En-Y gastric bypass procedures. | 1 |
| **Cholec80** | 80 videos of cholecystectomy surgeries performed by 13 surgeons. Frames are annotated for tool presence and surgical phases by a senior surgeon in France. | 23 |
| **Cholec120** | Contains 120 laparoscopic cholecystectomies from a single centre. | 1 |
| **CholecT45** | CholecT45 is a subset of CholecT50 and Cholec80. Contains 45 videos of laparoscopic cholecystectomy surgery annotated with 100 triplet classes (instrument, verb, and target). | 2 |
| **CholecSeg8k** | Based on the Cholec80, includes 8,080 extracted laparoscopic cholecystectomy stills from 17 video clips. Each frame is annotated for 13 surgical classes. | 1 |
| **EndoVis2017** | Part of the MICCAI Endoscopic Vision Challenge 2017, contains 10 video sequences of robotics surgery scenes. | 4 |
| **EndoVis2018** | Part of the MICCAI Endoscopic Vision Challenge 2018, contains 14 robotics surgery video sequences, depicting 8 surgical instruments. | 1 |
| **GBVasc181** | Contains 181 intra-operative images with manual contours (ROIs) of the gallbladder and 800 patches. Annotations were completed by expert surgeons. The images were extracted from Cholec80 video dataset. | 1 |
| **Hamlyn Centre video** | Consists of ~40,000 pairs of rectified stereo images collected in robotic-assisted partial nephrectomy using the Da Vinci. | 4 |
| **HeiCo** | Heidelberg colorectal (HeiCo) contains laparoscopic video data from 3 types of colorectal surgery, accompanied with the corresponding streams from medical devices. Annotations include surgical phase, and instrument presence for more than 10,000 frames. | 1 |
| **LapSig300** | Contains 300 laparoscopic colorectal surgery videos from 19 high-volume endoscopic centres in Japan. Annotated with surgical phase and actions. | 1 |
| **MICCAI 2015** | MICCAI Endoscopic Vision Challenge 2018 [27] dataset is a public dataset with 14 video sequences on robotics surgery procedures. | 2 |
| **MultiType** | Contains 80 different laparoscopic surgeries and accompanying surgical device data from 1 centre in Germany. | 1 |
| **M2cai16-tool** | Contains 15 videos, including spatial tool annotations for 2,532 frames across the first 10 videos. The dataset consists of 3,141 annotations of 7 surgical instrument classes. | 10 |
| **World Laparoscopy Hospital** | Free high-definition minimal access surgical procedures from India, Dubai and USA. | 1 |
| **SAGES YouTube** | Minimally invasive surgery videos contributed by Society of American Gastrointestinal and Endoscopic Surgeons (SAGES) members and presenters at annual meetings. | 1 |

**Appendix 4.** *Details of data annotation*

| **Characteristic** | **(n=122)** | | |
| --- | --- | --- | --- |
| Annotation software | The ANVIL Video Annotation Tool  Computer Vision Annotation Tool (CVAT)  Surgery Workflow Toolbox (B<>com Annotate)  Microsoft and Wacom Surface Pens  Custom designed  Image Labeller MATLAB  Other*  Not reported or N/A (unsupervised, unclear, or not specified ML) | | 6  3  3  3  3  3  7  94 |
| Number of annotators | 1  2  3  4  5  Not reported or N/A (unsupervised, unclear, or not specified ML) | | 10  26  7  1  8  70 |
| Annotator | Clincal personnel  Non-clinical personnel  Both clinical + non  AI generated  Not stated  Not reported or N/A | Surgeons  Trainees and students  Undefined medical personnel  Both surgeon + student | 34  8  4  1  4  2  1  2  66 |
| Experience/training of annotators | Use of protocol or guidelines  Clincial experience or expert knowledge  Supervision  N/A, or not reported | | 11  8  1  102 |
| Inter-rater/cross checking of annotations | Annotations validated by multiple experts  Statistical analysis to calculate agreement rates  Discrepancies solved via discussion  Likert scales  Not stated  N/A (unsupervised, or Machine learning)  Unclear | | 13  11  4  1  71  20  1 |

***** Other software includes 1 of each of the following: Think Like a Surgeon, Theator, Humanome Eyes Workstation, Real-time multi-display educational system (ARMES), Annotator - a Fiji plugin, Labe-lImg and Robofow web application.

**Appendix 5.** *Characteristics of machine learning and outcome measures*

| **Characteristic** | **(n=122)** | |
| --- | --- | --- |
| Method of machine learning | Supervised  Mix of both supervised & unsupervised  Unsupervised  Not reported  Unclear | 92  10  5  8  7 |
| Validation methods | Internal  Unclear  Not specified  External  Both internal & external | 93  11  8  6  4 |
| Use of comparator | No  Yes  Unclear | 83  35  4 |
| Type of comparator | Other state-of-the-art AI models  Other methods/techniques | 29  6 |
| AI undertaken in real-time | No  Yes  Unclear | 106  14  2 |
| Outcome measures* | Overall and best accuracy  Precision, recall and F1 score  Sensitivity  Predictive values  Specificity  Area under the curve (AUC)  Dice similarity coefficient  Intersection over union (IOU)  Jaccard score  Multiple (of the above)  Other**  Unclear  No measure of accuracy reported | 67  58  22  15  13  10  8  5  4  67  6  3  1 |

*Multiple outcome measures are reported therefore numbers will exceed total studies

** Other includes; 1 percentage of safer identification of landmarks, 1 automatic instrument detection time vs manual detection time (sec), 2 colour consistency score, 1 tracking robustness

**Appendix 6**. Accuracy of AI performance and reported limitations (total n=61)

| **First author and date** | **AI objective(s)** | **Accuracy (%, mean, standard deviation)** | **Reported limitations** |
| --- | --- | --- | --- |
| Luongo 2021 | Task and/or gesture identification | 62% (average) classification task | Limited training data |
| Sanat 2021 | Surgical phase/workflow recognition | Joint phase and step recognition  (best performance, mean±std)  89.1 ± 2.4 (no TCN)  91.2 ± 2.5 (MTMS-TCN stage 1)  90.9 ± 2.8 (MTMS-TCN stage 2) | - |
| Mascagni 2021 | Enhanced intra-operative visualisation | Average 88% | - |
| Shi 2020 | Surgical phase/workflow recognition | Full annotation = 85.73 (mean±std)  active learning = 85.87 ± 7.36 (mean±std) | - |
| Jin 2019 | Surgical phase/workflow recognition + Instrument recognition (incl. tracking) | 89.2 ±7.6 8 (mean±std) | Visual quality (obscured video frames) |
| Bamba 2021 | Instrument recognition (incl. tracking)  Identification of anatomy | 83% | Limited training data |
| Du 2019 | Instrument recognition (incl. tracking) | 79.01% with 20-pixel threshold | Limitations with model when distinguishing foreground and background |
| Derathé 2020 | Skill assessment | 0.68 | - |
| Kitaguchi 2020 | Surgical phase/workflow recognition | 91.9% phase recognition | Limited dataset (one centre) |
| Chen 2018 | Surgical phase/workflow recognition | 76.8% performance detection | Difficulties capturing complicated movements during the surgery in subtle scenes |
| Loukas 2015 | Smoke detection | ≥ 80% |  |
| Nakawala 2019 | Surgical phase/workflow recognition | 74.29% | Limited computational memory |
| Namazi 2022 | Instrument recognition (incl. tracking) | 85.77% and 91.92% Cholec80  80.95% and 81.84% M2CAI16 | - |
| Cheng 2022 | Surgical phase/workflow recognition | 91.05% | Limited dataset |
| Park 2023 | Enhanced intra-operative visualisation + Surgical phase/workflow recognition | 91% | Limited dataset (small number of patients from a single cohort) |
| Madani 2022 | Enhanced intra-operative visualisation | >90% | Limited dataset (only of ten frames, which represents a very small proportion of the total number of frames from each video). |
| Mascagni 2022 | Enhanced intra-operative visualisation | 71.4% (balanced accuracy) | DeepCVS makes predictions on still pictures showing an anterior (medial) view of the hepato-cystic triangle, not the doublet view suggested for photographic documentation. |
| Kitaguchi 2020 | Surgical phase/workflow recognition  Task and/or gesture identification | 81.0% surgical phase  83.2% action classification | Single video annotator |
| Loukas 2021 | Identification of anatomy | 94.48% (2 classes) patch classification  83.77% (3 classes) Gall Bladder wall regions  91.16% (2 classes) Gall Bladder wall regions  80.66% (3 classes) Gall Bladder wall regions | - |
| Dergachyova 2016 | Surgical phase/workflow recognition | 68.10% study 1  78.95% study 2  88.93% study 3 | - |
| Lavanchy 2021 | Instrument recognition (incl. tracking) Task and/or gesture identification | 87 ± 0.2% (mean±std) | - |
| Hashimoto 2019 | Surgical phase/workflow recognition | 82% ± 4% (mean±std) | Limited dataset (videos from single institution) and limited data annotation (limited to the major, straight forward steps of Laparoscopic Sleeve Gastrectomy, rather than focusing on more granular sub-steps or techniques). |
| Cai 2020 | Instrument recognition (incl. tracking) | 91.2% EndoVisSub dataset  75% standard dataset | Image blurring caused by the instrument's fast moving can lead to bad detection results, and our method is limited to a straight instrument. |
| Kitaguchi 2022 | Surgical phase/workflow recognition | 93.2% | Limited data (number of procedures and surgeons) and cross-validation was omitted in this study because there were no statistically significant differences in patients' characteristics between the training and test sets. |
| Lee 2020 | Skill assessment | 83% | Further training required on more varied surgical areas. Also, we could not directly compare the performances of the kinematics and proposed image-based methods because access to the Da Vinci Research Interface is limited, allowing most researchers only to obtain kinematic raw data. |
| Derathé 2021 | Skill assessment | 0.64 ± 0.05 Data cluster CESS  0.60 ± 0.08 Data cluster CSPP  0.66 ± 0.15 Data cluster CA ESS  0.73 ± 0.05 Data cluster CB ESS  Data cluster CA SPP  Data cluster CB SPP | - |
| Kannan 2020 | Identification of procedure type | 75% | - |
| Bar 2020 | Surgical phase/workflow recognition | >90% | Limited dataset (one specific procedure) |
| Meeuwsen 2019 | Surgical phase/workflow recognition | 77% | Lack of real-time phase recognition |
| Alshirbaji 2021 | Instrument recognition (incl. tracking) | 93.95% | variation in the frequent appearance of the surgical tools results in imbalanced data problem |
| Jin 2018 | Surgical phase/workflow recognition | 90.7% MICCAI Workflow  92.4% Cholec80 | - |
| Zhao 2019 | Instrument recognition (incl. tracking) | 93% in vivo dataset  82% cardiac dataset | The results of the cardiac condition show that our method has short comings. Firstly, the re-location operation by the CNN took a long time over the entire tracking process, and secondly, the relocation operation increased the number of failing frames. |
| Huaulmé 2020 | Enhanced intra-operative visualisation | >90% (best performance) | Limited dataset (manual annotations by one observer, surgeries performed by a single surgeon, and the dataset includes bleeding events only). |
| Khoiy 2016 | Instrument recognition (incl. tracking) | 97% for high quality images  80% for poor quality images | - |
| Baghdadi 2019 | Skill assessment | 83.30% | Limited dataset (data source from a single and limited by the small number of videos). |
| Padoy 2012 | Surgical phase/workflow recognition | >97% | - |
| Garcia-Martinez 2017 | Enhanced intra-operative visualisation | > 88% | - |
| Marullo 2023 | Enhanced intra-operative visualisation  Instrument recognition (incl. tracking) | 90.63% | It has been observed experimentally that the accuracy reduces considerably, particularly for segmentation masks, due to the rapid movement of the surgical instruments. |
| Jalal 2019 | Surgical phase/workflow recognition Instrument recognition (incl. tracking) | 93.1% (best performance) | Limited dataset (single dataset consitsting of Cholec80). Furthermore, the spatial and temporal models were trained separately, not end-to-end. |
| LAI 2023 | Enhanced intra-operative visualisation | 0.899 (average) | Relatively small dataset, and there were considerable false positive predicted bounding boxes during the detection process. |
| Zhang 2023 | Surgical phase/workflow recognition | 82.74% (best overall)  95.67% video-by-video | - |
| Kitaguchi 2021 | Skill assessment | 75.0% (mean)  83.8% (best performance) | Video clips extracted from the same surgery were included in both the training and test data sets. Therefore, the generalizability of the proposed model for unseen surgical procedures and surgeons could not be reliably established. Limited dataset (all videos were collected from Japanese hospitals, and the ESSQS by the JSESis not widely used worldwide for surgical skill assessment). |
| Loukas 2022 | Identification of anatomy | 92.6% - 93.2% (range) | Limited training data and manual delineation of the gallbladder ROI. However, given a larger dataset of gallbaldder images this burden could be alleviated via deep learning image segmentation techniques. multiple |
| Takeuchi 2023 | Surgical phase/workflow recognition | 75.4% | Limited dataset (single centre and procedures by one surgeon) |
| Choksi 2023 | Surgical phase/workflow recognition | 68.7% (average) | Limited dataset (single centre and procedures by one surgeon). Annotations by only one expert. |
| Ortenzi 2023 | Surgical phase/workflow recognition | 88.8% complete procedure | Unequal dataset (e.g. 74.5% of videos were collected from a single medical centre) |
| Tao 2023 | Surgical phase/workflow recognition | 93.12 ± 4.71% (average) | Limited dataset |
| Golany 2022 | Surgical phase/workflow recognition | 89% (mean) | Limited dataset (non-real-world data) and non-real time nature of the system, which does not allow it to be used to provide safety indications during the procedure. |
| Liu 2023 | Identification of anatomy | The accuracy of MRF-MASK model was at least 138.1% (median ODA) and 100.0% (mean ODA) higher than that of MHD model. | The proposed method concentrates on surface deformation tracking in 2D since the input is monocular laparoscopic video. |
| Eckhoff 2023 | Surgical phase/workflow recognition | 87.7 ± 7 (mean±std) | Limitations with accuracy of model (tendencies of confusing dissection phases, "Dissection Gastrocolic Ligament" and "Dissection Gastrohepatic Ligament). |
| Lo Muzio 2021 | Enhanced intra-operative visualisation | >75% highest classification models KNN and SVM | - |
| Ban 2024 | Identification of anatomy  Instrument recognition (incl. tracking) | 62.4% conceptNet-ResNet50  67.1% conceptNet-VIT | - |
| Park 2023 | Instrument recognition (incl. tracking) | 64.40±6.52 Slowfast50  48.39±3.67 Bi-LSTM  57.23±3.36 PerceiverIO (rawdata)  52.70±3.98 PerceiverIO (feature) | Quality of data (the model cannot recognize the organ and the instrument if the scene has blurry or bloody sections). |
| Takeuchi 2023 | Identification of anatomy | 77.1% (mean) | Limited dataset (single centre, no female patients and a limited number of cases). A second limitation is that the assessment of surgical videos (annotation) may lead to inconsistent annotations, because it is dependent on surgeons. |
| Cui 2021 | Identification of anatomy | 94.61% image dataset  92.93% video dataset | Limited dataset (number of patients, training and test data). |
| Sasaki 2022 | Surgical phase/workflow recognition | 0.891 model 1  0.947 model 2 | Lack of varied dataset and from a single institution. |
| Arpaia 2022 | Enhanced intra-operative visualisation | 99.9% | Future work will be addressed to overcome the current research weakness, by (i) introducing more levels between adequate and inadequate perfusion, in order to increase the resolution of the assessment and further enhance accuracy of prediction, (ii) identifying a method to automatically select the ROIs, and (iii) enrich the dataset by facing circumstances when the blood perfusion is impaired by underlying pathologies (e.g., atherosclerosis). In this case, in fact, both the classifer and the surgeon are not trained to correctly assess whether perfusion is adequate or not. |
| Kadkhodamohammadi 2022 | Surgical phase/workflow recognition | 93.77 ± 0.44 (mean±std) | Noisy encoder features or difficulty of using the long-term temporal context. |
| Pan 2023 | Surgical phase/workflow recognition | 92.8% | - |
| Das 2022 | Surgical phase/workflow recognition | Multiple accuracies reported refer to Table 1-3 in paper | - |
| Liu 2023 | Surgical phase/workflow recognition  Task and/or gesture identification | 0.82 steps  0.80 activities | Limited dataset (future research should focus on increasing the training sample size to improve the model's accuracy). |
| Van der Stel 2023 | Enhanced intra-operative visualisation | 90.9% | Thus, a limitation of this study is the observation that no anastomosis-related postoperative complications were recorded. |
| Demirel 2022 | Instrument recognition (incl. tracking)  Skill assessment | 95% no-tool classification  87% shaver tool  85% electrocautery tool | Model accuracy (e.g. when a tool in the frame is heavily occluded or mostly out-of-frame, the model's accuracy decreases because the defining features of the tools are not visible). Another limiting factor in the automation case study was training data. Manually defining the ground truth for the amount of time that a tool is present in a video is laborious and limits the size of the dataset. |
| Guzmán-García 2022 | Surgical phase/workflow recognition | 0.788 ± 0.089 (mean±std) video-based model  0.778 ± 0.157 (mean±std) speech-based model  0.819 ± 0.042 (mean±std) complete algorithm | The main limitation of this study corresponds to the high computing times that it currently conveys. |
